# Supplementary figures and images for: A non-canonical activation of the host’s ESCRT machinery is required for the scission of parasitophorous vacuoles and the replication of Leishmania donovani
Source: PLoS Pathog. 2025 Sep 16;21(9):e1013513. doi: 10.1371/journal.ppat.1013513 (PMC12453208; doi:10.1371/journal.ppat.1013513)

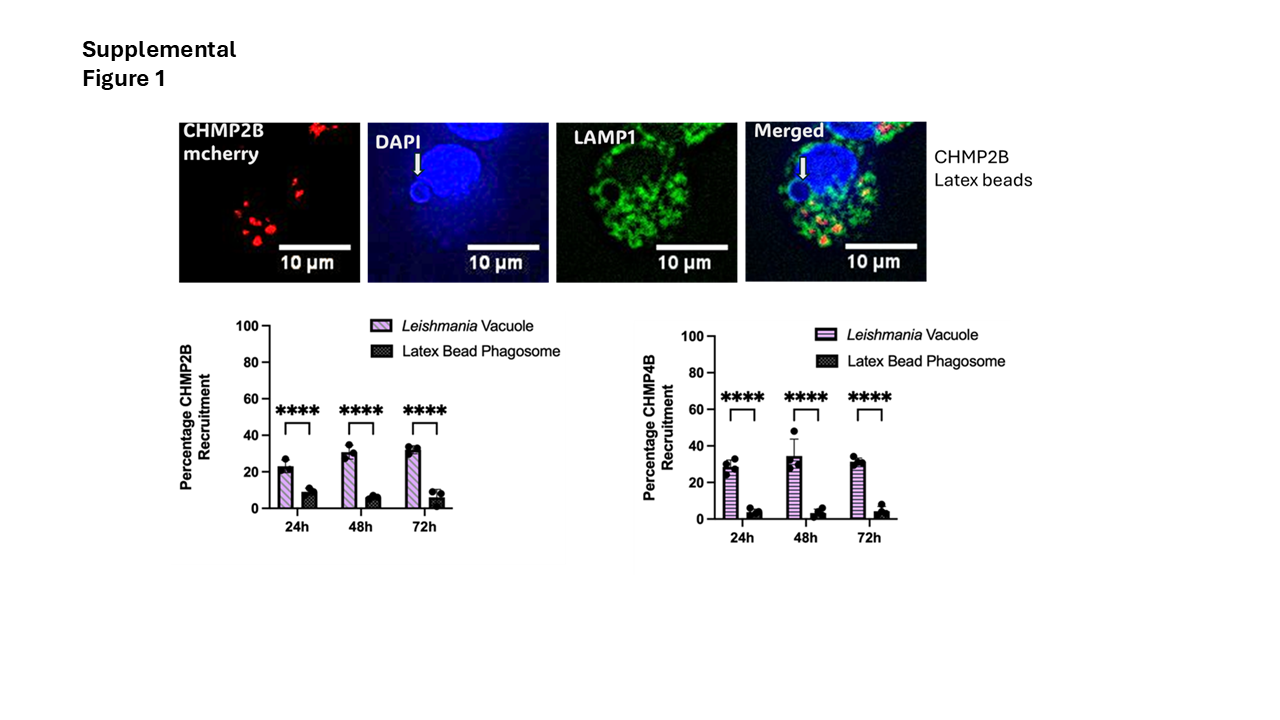

Supplement: S1 Fig — Recruitment of ESCRT components to generic phagosomes was determined by incubating transfected cells with latex beads. A representative image of a cell transfected with CHMP2B and a phagocytosed latex beads is shown. The proportion of phagosomes that displayed CHMP2B or CHMP4B is shown in the associated graphs. LAMP1 reactivity was used to delineate the vacuole of the LPV or latex bead phagosome. White arrow points to a latex bead. (TIF) [file ppat.1013513.s001.tif]

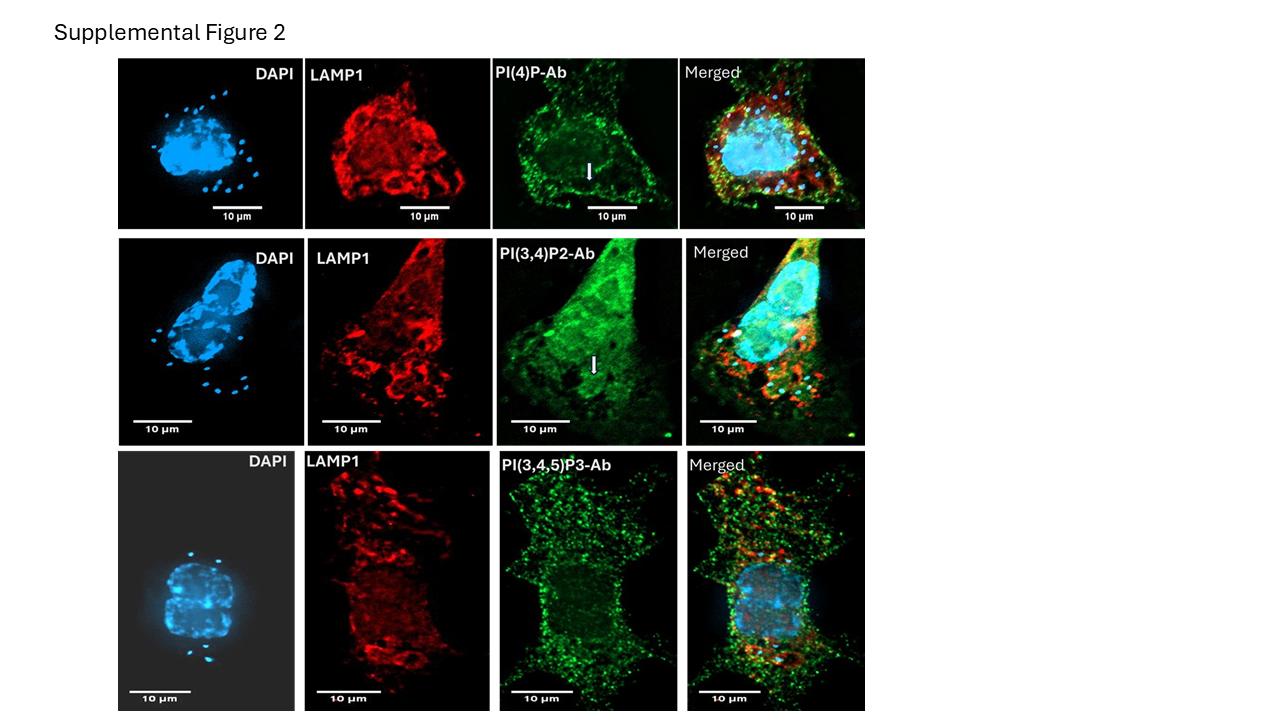

Supplement: S2 Fig — THP-1 cells on coverslips were infected with L. donovani. After 48 hours coverslips were fixed and processed for immunofluorescence detection of phosphoinositides with antibodies to each phosphoinositide. Representative images of THP-1 cells infected for 48 hours are shown. These images are representative of two experiments. (TIF) [file ppat.1013513.s002.tif]

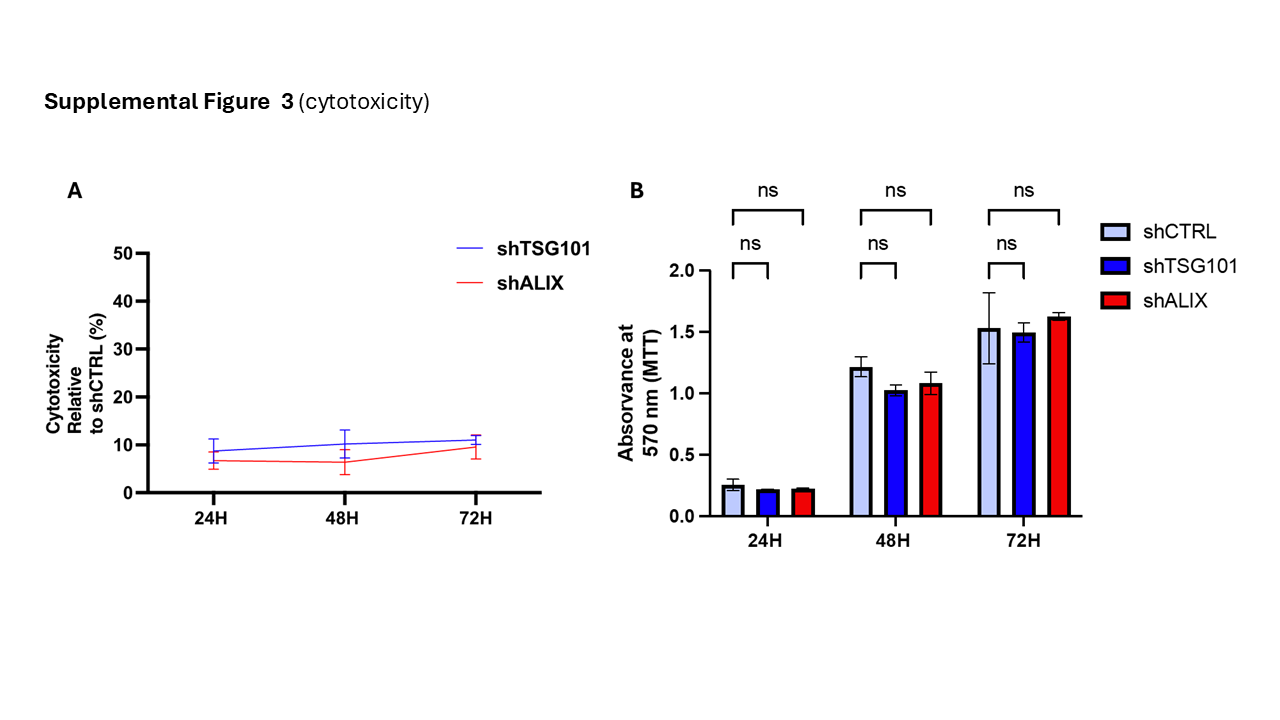

Supplement: S3 Fig — (A). Cytotoxicity was determined by monitoring LDH release from oligoclonal KD cells and from shCTRL. For the MTT proliferation assay, cells were seeded in 96-well plates, with 4 wells per cell line, and separate 96-well plates were used for each time point. (B) Proliferation was assessed using the MTT cell proliferation kit AR1156 (BOSTER). The results are from 2 experiments. (TIF) [file ppat.1013513.s003.tif]

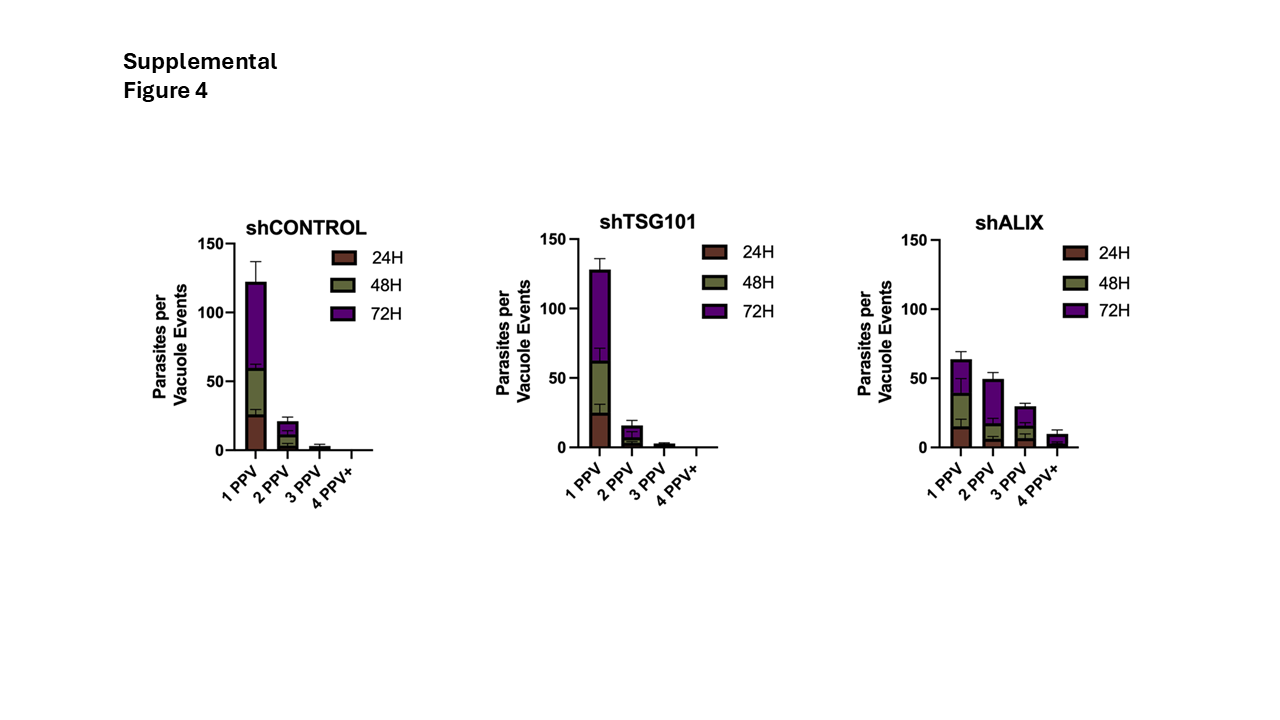

Supplement: S4 Fig — Infected cells on coverslips were fixed after 24, 48, and 72 hours of infection. Immunofluorescence labeling of LAMP-1 permitted detection of the contours of LPVs. Parasite nuclei were detected with DAPI. The number of parasites per LPV was counted at 24, 48, and 72 hours after infection. At least 100 LdLPVs were measured from each coverslip. Data was compiled from at least 3 experiments and graphed using GraphPad Prism 8. (TIF) [file ppat.1013513.s004.tif]

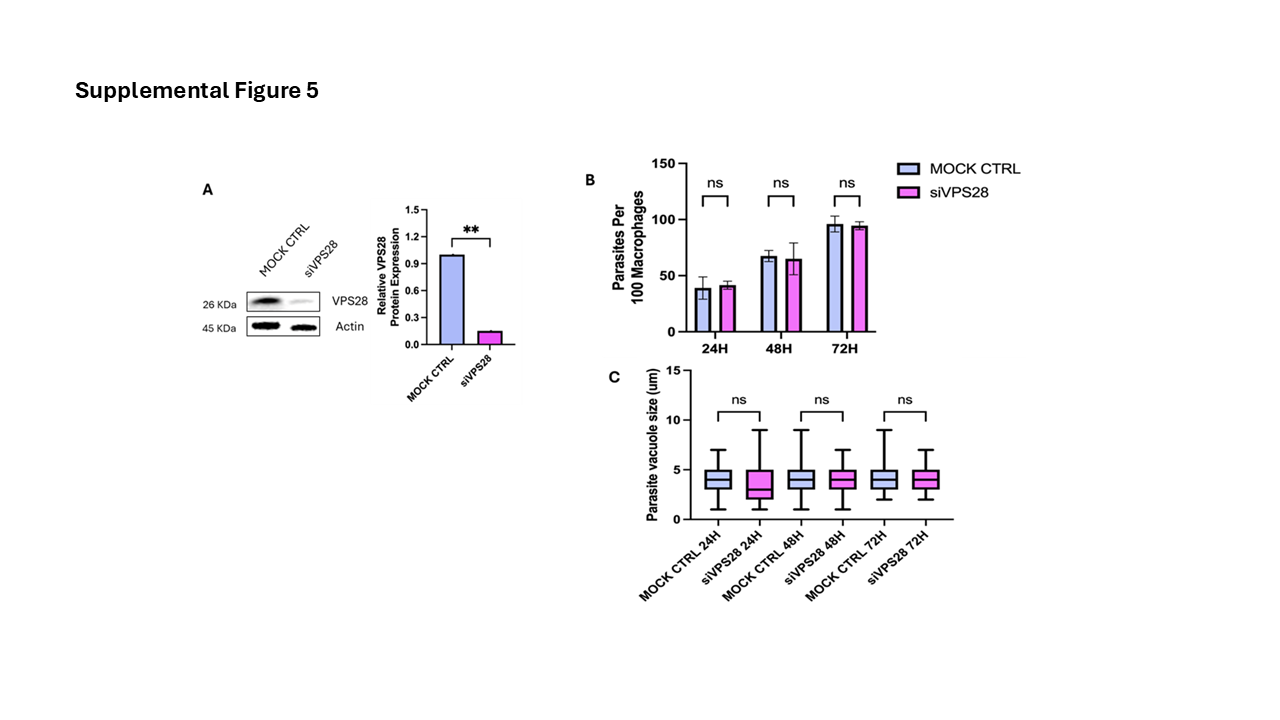

Supplement: S5 Fig — RAW264.7 cells were transfected with siRNA to VPS28 and plated in dishes with or without coverslips. Control cells were mock-transfected. Lysates were prepared from dishes without coverslips. Lysates were analyzed by Western blotting for VPS28 expression (A). Plot from the densitometric analysis is shown. B) Cover slips were infected with L. donovani parasites for 24, 48 or 72hs. Immunofluorescence labeling was performed to evaluate the infection. The number of parasites per macrophage in infected VPS28 KD cultures, as compared to infections in mock controls, was enumerated and plotted. Data was compiled and graphed using GraphPad Prism 8. C) LAMP1 labeling was performed to confirm LdLPVs. The LdLPV sizes in KD lines after 24, 48 and 72 hrs infection were measured and plotted. At least 100 LdLPV sizes were measured per coverslip. A one-way ANOVA was performed, and statistical significance was determined by post hoc Tukey’s honest significant difference test. For western blotting validation, an unpaired student T test was performed for statistical analysis. Data were compiled and graphed using GraphPad Prism 8. *p < 0.05, **p < 0.01, ***p < 0.001, ****p < 0.0001. N.S, not significant. (TIF) [file ppat.1013513.s005.tif]

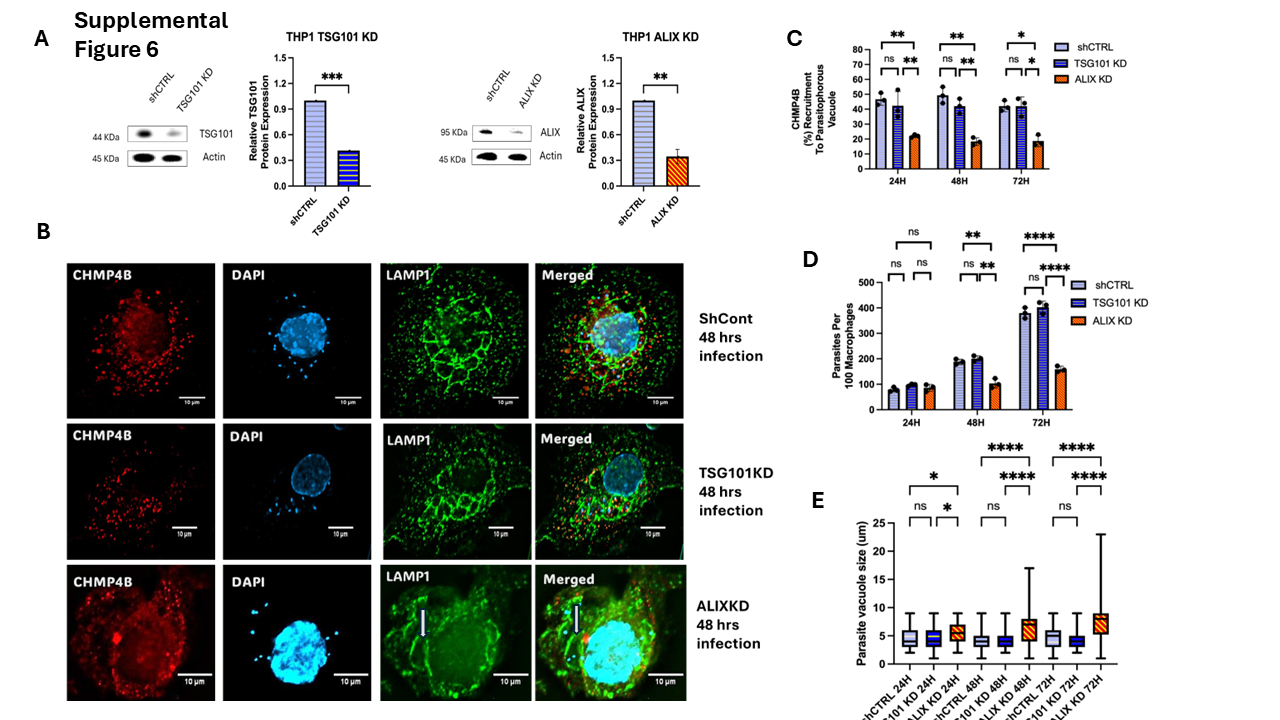

Supplement: S6 Fig — The TSG101 shRNA plasmid, Alix shRNA plasmid, and Control shRNA plasmid were transfected into THP-1 monocytes. Stably transfected cells were selected by growth in media supplemented with puromycin. Lysates from stable lines were analyzed in Western blots. A) Representative blots were probed for TSG101 or ALIX, and densitometric analysis of blots was plotted. B) The recruitment of CHPMP4B to LdLPVs in these cell lines was evaluated. Representative images of infected cells, labeled with CHMP4B (red), LAMP1 (green), and DAPI (blue), are shown. Also shown are the proportions of LdLPVs shCTRL, TSG101KD and ALIXKD that recruited CHMP4B. At least 100 LdLPVs were measured per coverslip, per time point, and treatment. C) The sizes of LdLPVs in shCTRL, TSG101KD, and ALIXKD were measured and plotted. At least 100 LdLPV sizes were measured per coverslip, per time point, and treatment. D) The number of parasites in infected shCTRL, TSG101KD, and ALIXKD were enumerated. At least 100 macrophages were counted per coverslip, treatment, and time point. A one-way ANOVA was performed, and statistical significance was determined by post hoc Tukey’s honest significant difference test. For western blotting validation, an unpaired student T test was performed for statistical analysis. Data were compiled and graphed using GraphPad Prism 8. *p < 0.05, **p < 0.01, ***p < 0.001, ****p < 0.0001. N.S, not significant. (TIF) [file ppat.1013513.s006.tif]

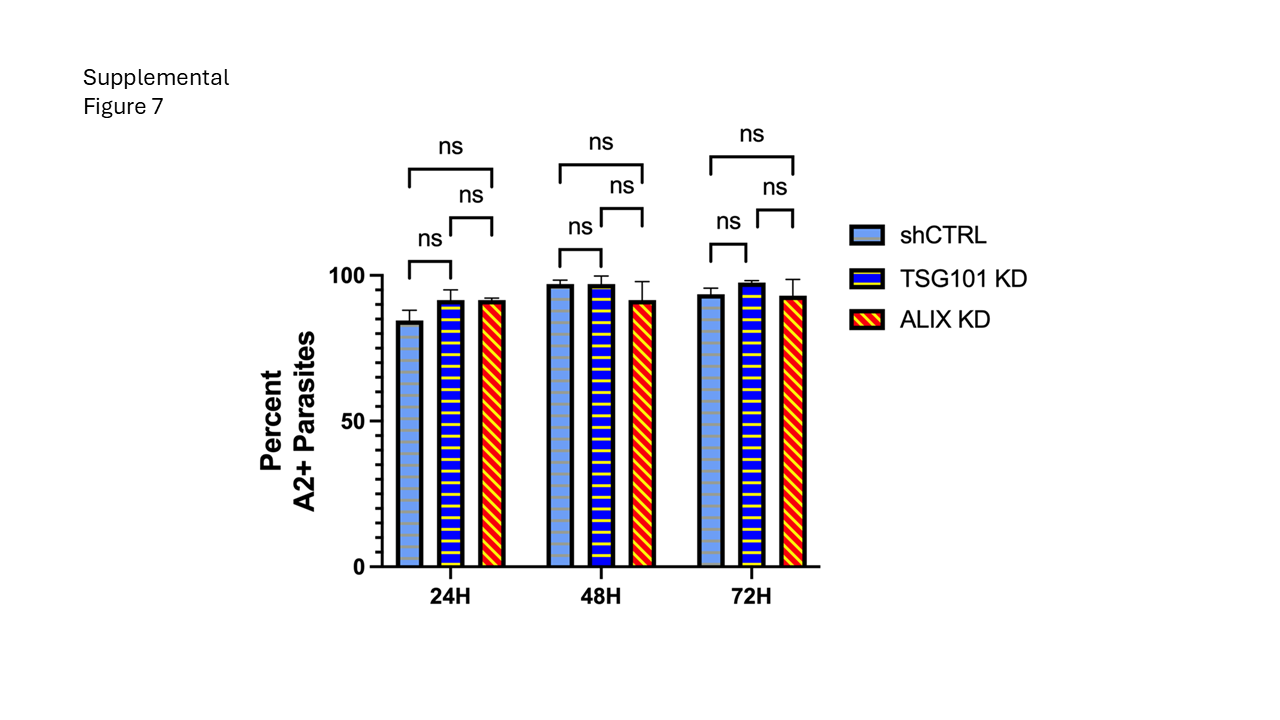

Supplement: S7 Fig — After the differentiation of THP-1 monocytes into macrophages, they were infected with infective metacyclic promastigote forms of L. donovani. At 24, 48, and 72 hrs post-infection, cells on coverslips were fixed and processed by immunofluorescence labeling for the detection of A2. At least 100 parasites were counted per coverslip, treatment, and time point. The percentage of parasites expressing A2 was compiled and graphed using GraphPad Prism 8. A one-way ANOVA was performed, and statistical significance was determined by post hoc Tukey’s honest significant difference test. Data was compiled and graphed using GraphPad Prism 8. *p < 0.05, **p < 0.01, ***p < 0.001, ****p < 0.0001. N.S, not significant. (TIF) [file ppat.1013513.s007.tif]

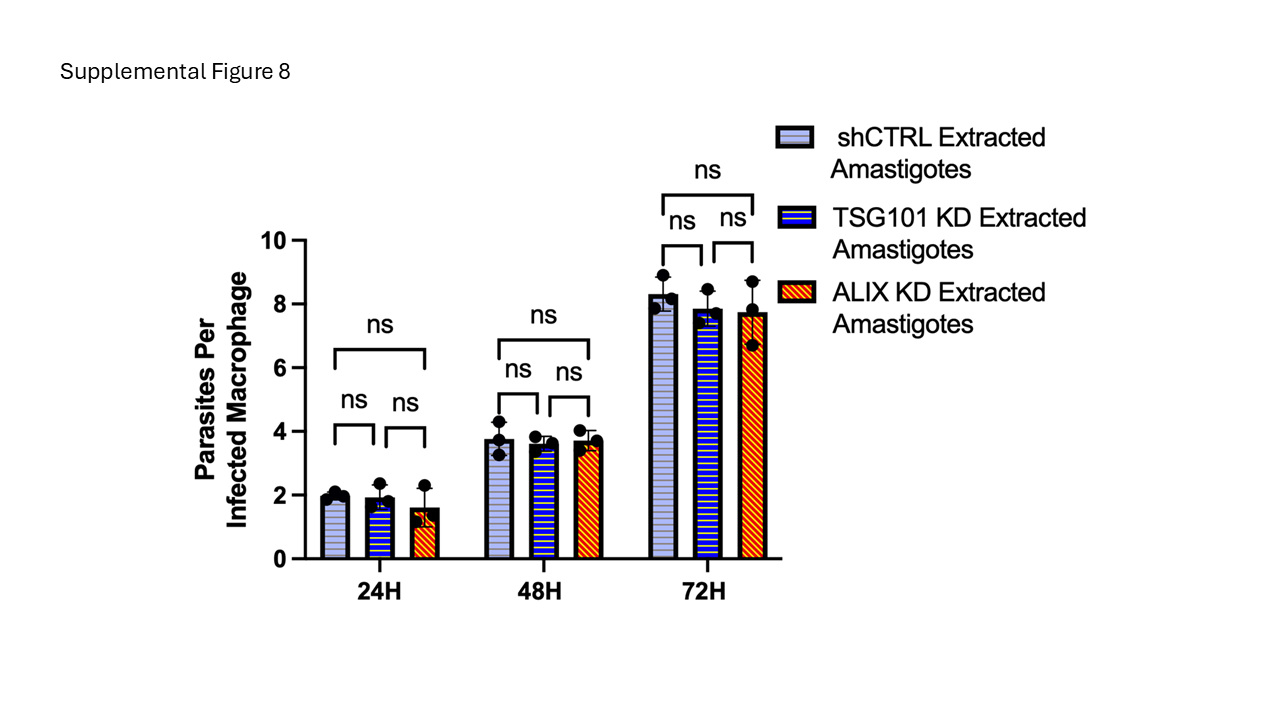

Supplement: S8 Fig — L. donovani amastigotes were recovered from shCTRL TSG101KD and ALIXKD THP-1 cell lines after 72 hours of infection. The parasites were counted and then added to THP-1 macrophages on coverslips in 6-well plates at a 5:1 parasite-to-macrophage infection ratio. Infections were scored at 24, 48, and 72 hours of infection. At least 100 macrophages were counted per coverslip in cells infected with recovered parasites. A one-way ANOVA was performed, and statistical significance was determined by post hoc Tukey’s honest significant difference test. Data was compiled and graphed using GraphPad Prism 8 *p < 0.05, **p < 0.01, ***p < 0.001, ****p < 0.0001. N.S, not significant. (TIF) [file ppat.1013513.s008.tif]
