# Supplementary material for: A non-canonical activation of the host’s ESCRT machinery is required for the scission of parasitophorous vacuoles and the replication of Leishmania donovani
Source: PLoS Pathog. 2025 Sep 16;21(9):e1013513. doi: 10.1371/journal.ppat.1013513 (PMC12453208; doi:10.1371/journal.ppat.1013513)
Supplement: Attachment — Submitted filename: Response to reviewers for plos pathogens paper.doc [file ppat.1013513.s011.doc]

Department of Microbiology and Cell Science	Room 1052 Building 981

PO Box 110700

Gainesville, FL 32611-0700

(352) 392-1906

Fax: (352) 392-5922

We thank the Reviewers for critically reviewing our manuscript and for providing constructive suggestions on how to improve the manuscript. Please find below specific and detailed responses to their criticisms and suggestions.

'Response to Reviewers'

Reviewer #1 wrote: *Rosero and Kima report on the role of the ESCRT machinery in the scission of parasitophorous vacuoles containing the parasite Leishmania donovani (LdLPVs) as well as in the replication of the parasite. They found that ESCRT components are constitutively recruited to LdLPVs, in a PI(3,4)P2-dependent manner. Knockdown of ALIX led to a significant reduction in parasite burden and replication and to an increase in LdLPV size. Overall, this is an interesting study which addresses an important question in the cell biology of intracellular infection, namely how do individual vacuoles replicate at the same time than the pathogen they harbor. However, a number of issues need to be addressed to strenghthen the conclusions of this study.*

Response: We thank the Reviewer for a clear and concise summary of our study and for their assessment that ‘this is an interesting study which addresses an important question in the cell biology of intracellular infection…’ In the responses below, we will address the issues that the Reviewer raised.

*Reviewer #2: Rosero and Kima provide insight into the role of ESCRT in Leishmania biogenesis. The authors have observed and quantified the recruitment of several of the ESCRT components and then used shRNAs and dominant negative overexpression to manipulate the ESCRT pathway to demonstrate the role of ESCRT in parasite replication. The KD of ALIX prevents the scission of LdLPVs and multiple parasites buildup into the compartment with the absence of LdLPV division.*

Response. We thank the Reviewer for a succinct summary of our experiments and findings.

Reviewer #3: *The provided manuscript evaluated the role of ESCRT machinery in Leishmania donovani- infected macrophages. L. donovani invade macrophages, and another immune cells, as host cells, where they occupy a parasitophorous vacuole (PV) to differentiate and replicate. Bacteria that invade host cells have developed secretion system to introduce their proteins into the host cell, but no such system has been found in Leishmania spps. During replication within the host cell, the entire PV is split as well. The authors suggested that the host ESCRT machinery is involved in this process. They evaluated the role of ESCRT I protein TSG101, and ESCRT I accessory protein ALIX, seeing that ALIX was necessary for proper division of the PV after parasite replication. KD of ALIX resulted in PV containing multiple parasites. ESCRT III protein CHMP2B and CHMP4B were implicated in the division, and their recruitment to PV was used as a measure of typical ESCRT pathway function. When CHMP4B recruitment was reduced (because of the ALIX KD), this followed by a reduction in vacuole size, division, and parasite infectivity. I agree with the authors on their conclusions about the non-conical activation of these ESCRT proteins based on the data shown. The authors also implicate PI(3,4)P2 as being the phosphoinositide to play a role in recruiting ESCRT machinery. I don’t think this specific PI, or any PI, can be implicated from this data as presented.

I recommend this paper to be accepted with minor revisions. Clarification of the methodology behind the experiments included in Figure 3 is required to evaluate the conclusions made about PI(3,4)P2. Clarification in the formatting of the figures/images, and the corresponding text will make the conclusions regarding the localization of ESCRT proteins and the role of ALIX clearer to readers. Overall the data and methodology are sound, but some of the descriptions of the images are a bit difficult to follow.*

Response: We thank the Reviewer for a detailed summary of the rationale behind our studies and for their strong affirmation of the results and conclusions we reached. We noted that the Reviewer stated that they recommend that the paper be accepted. The Reviewer also said that there are experimental findings that they consider less supported by the experimental data. We welcome the opportunity to clarify our findings and to make changes to the manuscript that are consistent with the Reviewer’s suggestions.

Major concerns by Reviewers

*Reviewer #1: 1- An issue that remains to be addressed is why L. donovani fails to replicate in larger vacuoles observed in ALIX knockdown (KD) cells. In addition to affecting vacuole scission, it is possible that ALIX KD alters trafficking to and from the vacuole. It could be that ALIX is required for the acquisition of host factors essential for parasite growth. This issue may be addressed using L. mexicana complex parasites which thrive in spacious vacuoles. The authors should include these parasites to determine whether their replication is affected in ALIX KD cells*.

Response: We thank the Reviewer for bringing this issue to our attention. It is known that *Leishmania* species differ in some interactions within mammalian cells. *L. donovani* as compared to *L. mexicana* parasites establish vacuoles that exhibit different characteristics: *L. donovani live in individual vacuoles that divide when parasites divide; in contrast, L. mexicana* live in communal vacuoles. The Reviewer raised the possibility that the ESCRT machinery, or specifically ALIX may play a role in ‘acquisition of host factors essential for parasite growth’. The Reviewer suggested that infections with *L. mexicana* parasites should be evaluated in the ALIXKD cells that we described in the manuscript. We have proceeded to perform these experiments. Specifically, we infected THP-1 control transfected cells as well as THP-1 TSG101KD and ALIXKD cells with *L amazonensis* parasites. Following two biological repeats deemed successful, we observed no difference in the infection of L. amazonensis in ALIXKD cells compared to control KD cells or TSG101KD cells. Below is a figure generated from this experiment. Figure 1A shows the number of parasites per 100 macrophages at 24, 48, and 72 hours post-infection. In each cell line, there was a comparable increase in the number of parasites. In Figure 1B, we observed a similar rise in parasite vacuole sizes in all 3 cell lines**.** We interpret these results to mean that the factors that affect *L amazonensis* growth in macrophages is different from the factors that affect *L. donovani* growth in macrophages. In the manuscript, we showed that the knockdown of ALIX results in a limitation of *L. donovani* replication, which was linked to defective parasitophorous vacuole division.

**Figure 1. *L. amazonensis* infection is comparable in ALIXKD, TSG101KD and THP-1 transfection control cells.**

THP-1 (wild type) cells, ALIX and TSG101KD were infected with *L. mexicana amazonensis* (*L. amazonensis*) parasites. After 24, 48 and 72 hours cells on coverslips were fixed and processed analysis of the infection. A) DAPI labeling permitted the identification of parasite and host nuclei. Parasites in macrophages were enumerated at the indicated times after infection. B) LAMP-1 labeling allowed visualization of parasite-containing vacuoles (LdLPVs). The sizes of parasite vacuoles were determined at the indicated times. Counts were performed in triplicate on coverslips for 2 independent experiments. One-way ANOVA with multiple comparisons followed by *post hoc* Tukey’s honest significant difference test to determine statistical significance was performed. *p<0.05, **p<0.01, ***p<0.001, ****p<0.0001. N. S, not significant.

The growth requirements within infected macrophages may be different for *L. mexicana amazonensis* and *L. donovani*. Please bear in mind that in addition to harboring many *L. amazonensis* parasites within communal vacuoles, those vacuoles are spacious. This contrasts with the vacuoles observed in ALIXKD after *L. donovani* infections. Although the vacuoles in ALIXKD harbor increasing numbers of parasites, they are not spacious vacuoles. We believe that this data strengthens the observations in the manuscript; however, we have decided not to include this data in the manuscript. We believe that conducting more experiments to assess the recruitment of ESCRT components to *L. amazonensis* LPVs, including in the RAW264.7 macrophage cell line, is necessary before making strong statements on the role of the ESCRT machinery in *L. mexicana* spp. infections.

2- *Figure 1F, the authors show that overexpression of both CHMP4B and VPS4Adn in RAW cells significantly increased their recruitment to LdLPVs. It would be of interest to compare the growth of L. donovani in control RAW cells and in the CHMP4B/VPS4Adn RAW cells overexpressors*.

Response: We thank the Reviewer for making this suggestion. We had that data available when the original manuscript was submitted. We omitted to include it in the manuscript. This data has been included in the new Figure 2D.

*Reviewer #2: The authors make too strong of an argument for PI(3,4)P2 based on their immunostaining. The antibody (Z-P034) used does have a high enough specificity to rule out PI(4)P for example. Further the authors report in the Materials and Methods section the use of 0.5% Triton X-100 where the manufacture’s protocol warns against exceeding 0.2% Triton X-100 to prevent extraction of lipid populations. While the signal is likely PI(3,4)P2 it could be validated with PI binding proteins fused to GFP which have been well characterized. Further some to the real signal me be extracted with the high Triton X-100 concentration. Please consider mentioning the limitations or directly address the shortcomings with additional experiments.*

Response: We thank the Reviewer for this critical assessment of our findings on PI(3,4)P2. In this manuscript, as well as in a previously published manuscript (Zhang et al, 2018), we conducted several experiments to evaluate the spatial distribution of phosphoinositides in *Leishmania*-infected cells. In some experiments, cells were permeabilized with Triton X-100. In others, cells were permeabilized with saponin (the choice of permeabilizing agent was varied with consideration of other molecules and immune reagents of interest in those experiments). Consequently, we have experiments in which labeling of phosphoinositides was performed with permeabilization with saponin. In this revised manuscript, the section on phosphoinositides has been revised. We proceeded to track the distribution of the phosphoinositides in infected cells using both biosensors (as recommended by the Reviewer) and by antibody labeling after permeabilization with saponin. In the new Figure 4, we present representative images of cells labeled with the biosensors. We have also included representative images from antibody labeling in Supplemental Figure 2. We state in the manuscript that our objective was to affirm previously published studies that had investigated the spatial distribution of phosphoinositides in greater detail (Zhang et al, 2018). Moreover, we state in the revised manuscript that, equipped with these observations, we propose that PI(3,4)P2 is the likely anchor of ESCRT components to LdLPVs.

*Reviewer #2. The work would strongly benefit from electron micrographs (or even super resolution imaging may suffice) of the ALIX-KD to better document the alterations of the parasites and the LdLPVs, however this is not required for publication*.

Response: We thank the Reviewer for the suggestions that super-resolution imaging and electron microscopy could shed more insight into these interactions. We are pleased that the Reviewer stated that those approaches are not required for this publication. The role of phosphoinositides in this infection, including their biogenesis and distribution in infected cells, is a topic of interest to my laboratory. In future studies, we will utilize all available resources to gain further insight into the role of these molecules.

*Reviewer #2.* *The viability and parasitic health is not well established in the paper; if possible/practical please demonstrate the viability and infectivity of the parasitic progeny from the KD, DN and drug treated conditions.*

Response: We thank the Reviewer for raising this issue. In the manuscript, the Brdu experiments were one approach to address parasite viability. Loss of Brdu labeling would occur when parasites replicate. We showed that in control KDs and TSG101KD, parasites lost BrdU labeling between 24 and 72 hrs of incubation. In the ALIXKD, a significant number of parasites retained Brdu labeling during the same time frame. We reasoned that the retention of the label demonstrated that cells were viable but did not replicate. An additional assessment of parasite viability involved evaluating gene expression by the parasites. Specifically, we assessed the expression of A2 (Supplemental Figure 7). A2 is only expressed by viable parasites within macrophages. We showed that A2 expression was comparable in parasites within all knockdowns. Those studies notwithstanding, we have conducted new experiments that address parasite viability. Parasites were recovered after 72 hours of infection from control KD, ALIXKD, and TSG101 KD cells. The recovered parasites were used to infect new THP-1 macrophages with identical parasite-to-macrophage ratios. The infections were then scored after 24, 48 and 72 hrs. The results show that parasites recovered from shCTRLKD, TSG101KD and ALIXKD were equally infective (Supplemental Figure 8)

*Reviewer #3: Major Issues:

“The phosphoinositide PI(3,4)P2 on LdLPVs may be the target for recruitment of ESCRT molecules”

I’m confused about the conclusion being drawn here. The title for this section says that PI(3,4)P2 may be the target for ESCRT protein recruitment. PI(3,4)P2 is present on both the vacuoles that surround the latex beads and the Leishmania parasites, meaning it could just always be associated with phagocytosed particles, and not specific to Ld. The authors state they wanted to confirm that PI(3,4)P2 is present on the LdLPV because it has been shown to recruit ESCRT II. Images of infected cells stained with an antibody for PI(3,4)P2, show that it is recruited to LdLPV, and that recruitment is diminished when cells are treated with a general inhibitor of phosphoinositide synthesis (LY294002). They measure decreases CHMP4B. The conclusions is that PI(3,4)P2 is a target of ESCRT, and later in the discussion mention ALIX as the following step in recruitment. I think this is an overstatement, I don’t think this data definitively shows that PI(3,4)P2 is responsible for recruitment of subsequent ESCRT proteins. 1) The reduction in CHMP4B recruitment is not statistically significant (or it’s not stated to be, there is no p value listed so I assume it is not significant, but may have been a mistake). I don’t think the conclusion that PI(3,4)P2 is involved in ESCRT recruitment is supported if that reduction is not significant. 2) The authors haven’t shown that other phosphoinositides aren’t playing a role. The inhibitor used is a broad inhibitor, and there could be may downstream trafficking effects, which is stated in the discussion (long term treatment will kill the parasites by blocking one of their mechanism of evading macrophage activation). Evidence that other phosphoinositidies are not recruited/disrupted, and evidence that the antibody that they’re using is truly specific to PI(3,4)P2 and no other phosphoinositide is needed to draw this conclusion. If all phosphoinositides are disrupted, then even in the absence of PI(3,4)P2, if others are also absent, then they could be responsible for the phenotype. 3) To connect this phosphoinositide to ALIX in the discussion is a stretch, as only CHMP4B recruitment is measured. While I agree that it’s a reasonable hypothesis, there isn’t data presented here to support that conclusion.*

Response: We understand the source of the Reviewer's confusion. The figure of the *Zymosan* vacuole included was intended to demonstrate that *Zymosan* (inert particles) containing vacuoles do not display PI(3,4)P2 (as previously shown in Zhang et al., 2018). Unfortunately, the brightness of the image led to the impression that the phagosomes were positively labeled. Hence the confusion of the Reviewer. It was indeed the case that treatment of cells with LY294002 did not cause a statistically significant reduction in PI(3,4)P2. So that experiment was not conclusive. In this revised manuscript, edited that section of the manuscript and limited our reliance on that figure. As an aside, we are now aware of publications that have reported that the PI3KC2, which is the likely kinase that catalyzes the conversion to PI(3,4)P2 is insensitive to the pan PI3kinase inhibitors, including LY294002.

*Reviewer wrote: There is a more nuanced discussion on this in the discussion, so I believe the authors understand the complexities of these interactions, but I think that nuance isn’t translated in the explanation of the results, or the following sentence from the discussion:
“Our studies revealed a chain of events that commences with the display of the phosphoinositide, PI(3,4)P2 on LdLPVs, followed by the recruitment of ALIX to LdLPVs.”
To summarize, I think the statistical significance of 3B needs to be clarified. Experiments showing that this chemical blocker only effect PI(3,4)P2 recruitment and not any other PI are required, or the language could be modified to not overstate the role of PI(3,4)P2

PI The inhibitor you used can inhibit the synthesis of other phosphoinositides, and therefore other forms in other organelles could be playing a role in ESCRT recruitment. I think this sentence, as written, suggests you had data that showed that ALIX is recruited in response to PI(3,4)P2, however you measured only CHMP4B (ESCRT II) recruitment. While I agree with your later points that ALIX plays a more essential role than TSG101 on the ESCRT I front, and KD of ALIX led to less CHMP4B recruitment, so it’s a reasonable hypothesis, but I think it’s overstating to say that PI(3,4)P2 is followed by ALIX from the data present in this paper.*

Response: We thank the Review for their well stated concerns about the phosphoinositide experiments and how the experiments were discussed. The title of the section has been changed to ‘the phosphoinositide PI(3,4)P2 on LdLPVs may be the target for recruitment of ESCRT molecules’. We present the results of studies with biosensors that detect phosphoinositides. We also included new images from an immunofluorescence assay using antibodies. We have excluded the studies that used a pan-inhibitor of PI3 kinases.

**Part III – Minor Issues: Editorial and Data Presentation Modifications**
Please use this section for editorial suggestions as well as relatively minor modifications of existing data that would enhance clarity.

*Reviewer #1: 3- Figure 4 and Figure 6 are very similar. Whereas data shown in Fig 4 were obtained with RAW cells, data shown in Fig 6 were obtained with THP-1 cells. Figure 6 may be moved to the Supplemental data section*.

Response: We thank the Reviewer for that suggestion. In the revised manuscript, the old Figure 6 is now Supplemental Figure 6

*Reviewer 1- 4- Bottom of page 8, the authors state that "there was some increase in the frequency of LdLPVs that harbored more than 2 parasites (not shown)". Authors must show the data or remove this sentence.*

Response: We thank the Reviewer for raising this issue. We had the data when the manuscript was originally submitted. It is now included in Supplemental Figure 4.

*Reviewer 1- 5- Figure 4D and 4G, the authors state in the text (page 9) that "such larger LdLPVs harbored more that 4-10 parasites". The authors must quantify the number of parasites in the larger vacuoles in ALIX KD cells.*

Response: The data requested by the Reviewer is now included in the new Supplemental Figure 4.

*Reviewer 1 6- The authors must indicate at which time points post-infection were taken the images shown in Figures 1B, D, F, 4D, 5A, 6B.*

Response: Following the recommendation of the Reviewer, the times post-infection when the images were taken have been included in all figures.

*Reviewer 1 - 7- On several figures, font size must be increased to improve legibility.*

Response: We appreciate the Reviewer's suggestion. We have modified all images and included more legible captions.

*Reviewer #2: The paper is well written and moves the field forward.*

Response: We thank the Reviewer for this strong endorsement of our manuscript.

*Reviewer #3: Figure formatting: In the way this was submitted, all of the figures were not formatted clearly.
Figure 1 is split across 3 pages in it’s current form.

Stretched/Squished text that is difficult to read: Figure 2 graphs C-F, Figure 4 graphs E-G
Diagrams with small text/stretched that make it difficult to read: Figure 1A and E, Figure 2A, 4 A and D, Figure 5 A and B, and Figure 7A

Also, some of the graphs have titles and some don’t. Where there are titles, I think they’re a little confusing (ex, Fig 2 C-F). Can they be simplified or removed?*

Response: We thank the Reviewer for raising the issue of the non-uniformity of the Figures. The Figures have been reconfigured and or redrawn. It should be noted, though, that the nature of some experiments necessitates multiple figures. Consequently, some Figures are now in Supplemental Figures. This is especially necessary to minimize presenting squished or stretched texts, which the Reviewer targeted in their comments. Titles on graphs have been removed.

*Reviewer 3: The text in Figure 6 seems consistent in sizing and is clear to read, not blurry, and straight. Can be used an example*.

Response: We thank the Reviewer for the kind words concerning Figure 6 (Figure 7 in the revised manuscript). We have proceeded to modify the remaining figures accordingly. We have dropped some figures that do not improve the clarity of the manuscript.

*Reviewer 3: For all images: Need scale bars. Each individual fluorophore should be showed in white, and color only used in the merge, especially because the colors switch for LAMP1 depending on the fluorophore of each ESCRT protein. Also, the colors can be changed so they are consistent in merge (LAMP1 can be made red in all of the merges, whether you “collected” it as red or not, as long as all fluorophores are listed)*

Response. We have followed the suggestions of the Reviewer and proceeded to include scale bars to all images. Regarding the display of the fluorophores, we have chosen to present only the colored images for uniformity. In Figures where LAMP1 was detected with different fluorophore colors in order to accommodate the fluorophores of the biosensors, we have proceeded to use a single color for LAMP1, for clarity. The molecule that is been detected by each fluorophore is now clearly labeled.

*Reviewer 3. Introduction
“Leishmania donovani is the causative agent of visceral leishmaniasis that has an estimated 50,000 to 90,000 new cases per year, 95% of which are fatal if left untreated (https://www.who.int/news- room/fact-sheets/detail/leishmaniasishttps).”
Direct links to webpages should not be cited, the original source material should be cited*

Response*:* The webpage has been removed. Two references with the same information have been substituted here.

*Reviewer 3: “Interestingly, LPVs that harbor L. donovani (LdLPVs) acquire late endosomal characteristics slowly, as evidenced by their prolonged retention of Rab5 among other early endosomal molecules [6]. “
The citation of retention of Rab5 is incorrect, cited paper doesn’t reference Rab5 at all. Also this sentence starts with saying LdLPVs acquire late endosomal characteristics, but then you mention Rab5, an early endosomal marker. So I’m assuming the authors mean to say acquire early endosomal characteristics?*

Response: We thank the Reviewer for bringing to attention that the statement and paper cited in that statement were incorrect. We have edited the statement, but kept the citation, which does discuss the slow maturation of parasitophorous vacuoles that are initiated with *Leishmania donovani* metacyclic promastigotes

*Reviewer 3: Figure 1

The figure 1A does not match what is said in the text. Also, LAMP1 is written as all uppercase in the text, but all lowercase in the figures, this should be made consistent across the entire paper.

At least 1 outline/zoom in of the LdLPV would be helpful for each image, with arrows pointing to relevant features, (like what is seen in Figure 5). It’s hard to understand is being calling boundaries and periphery. Additionally, the explanation of CHMP2B and 4B forming a “neck” of the horn while the rim (LAMP1) is wider is not clear to me from these images. Looking at the merged picture for CHMP4B infected, in the LPV on the bottom right corner, CHMP4B and LAMP1 seem to have complete overlap of each other where they label together, while in the LPV directly to the left/slightly above the nucleus looks to have CHMP4B in more localized spots compared to LAMP1. I think an outline and arrows would be helpful to more clearly explain your point here, or a diagram, similar to Figure 5. I will say the image from supplemental 1 is much clearer than the image included in figure 1. To me, it seems there is a lot of similar colocalization of LAMP1 and CHMP4B, 2B, and Vps4A even in the uninfected cells, so the latex bead experiment is a great response to that for CHMP2B and 4B. Was this same experiment repeated for TSG101 or Vps4A? If so, the data should be included or mentioned.*

Response: We thank the Reviewer for the comments about Figure 1. We have determined that the key takeaway from the Figure is that ESCRT molecules are recruited to LPVs. Consequently, we have dropped that detailed description of the distribution of ESCRT molecules in these infected RAW264.7 macrophages. The results with THP-1 cells in Figure 7 are much more persuasive.

*Reviewer 3:The Vps4A data in 1b and 1c is not mentioned at all in the text, and that also looks like the colocalization is the same in infected vs uninfected. The overexpression experiment is clear and the conclusion makes sense, but I think the previous data needs to be mentioned/explained.*

Response: In the revised manuscript, the VPS4a data in the new Figure 2, is now discussed in the text.

*Reviewer 3. For 1F, the text states that a dual transfection of the Vps4 mutant and CHMP2B was used, and then the image and following sentences mention CHMP4B, so I assume 2B is a typo*.

Response: We thank the Reviewer for bringing this error to our attention. Yes. It was a typo. Although CHMP2B was also evaluated in those studies, we elected to show the CHMP4B results (new Figure 2) .

Reviewer 3: *For 1g, it is not mentioned in the text. Is this measuring recruitment of both CHMP4B and VPS4? Or some kind of ratio (given that the titles is “CHMP4B/VPS4Adn”)? The figure legend only mentions VPS4.*

Response: In this Figure, (now Figure 2), the recruitment of CHMP4B is evaluated in co-transfected cells that are also expressing VPS4dn. The Figure caption is simply to emphasize that this experiment is different from the experiments in Figure 1

*Reviewer 3. See notes for “Figure Formatting.” Can the individual points for 1D and 1G be added as in 1C?*

Response: Those figures have been re-formatted as suggested by the Reviewer.

*Reviewer 3. Figure 2
Overall this experiment is clear, as is the data. It seems that the CHMP4B is more localized within the LPV, rather than on the periphery as detailed in Figure 1b. How is LLOMe-mediated damage repaired? Does the ESCRT machinery associate and pinch off the damaged membrane to be sent to the plasma membrane? In which case, you’d expect to see CHMP4B on the outside of the PV, not inside. Do you have any explanation for what CHMP4B could be doing, and how it is getting inside the PV? Perhaps more appropriate for the discussion section, but a more detailed of how LLOMe-damage is typically repaired would strengthen the conclusions.*

Response: We thank the Reviewer for the comments about the LLOMe experiments. What we discovered from these experiments was that LLOMe induced damage and ESCRT mediated repair was mechanistically different from the involvement of ESCRT in Leishmania infections. After further investigations it is evident that the LLOMe damage story is well beyond the scope of this paper. A couple of references have been cited in the discussion section to guide anyone with further interest in this topic.

*Reviewer 3: For the figure legend in 1, you list as A) description. B) description. In the figure legend here, you list description (A), description (B). Pick one format and be consistent. Can each individual point be added to 2D and F as seen in Fig 1C?*
 *See notes for “Figure Formatting.”*

Response: The Figure legends have been made uniform.

*Reviewer 3: Figure 3
Why are two different time points used in this experiment (48 hours in no drug versus 24+8 in the treated)? The method section says 48 hours of infection and then 8 hours of treatment, which is not what the figure legend says. If you have images of the vehicle control, this would be a more appropriate comparison.

Most points were already mentioned in “major revisions” but another point to make is the change in PV morphology. It seems that PI is on the leishmania, but doesn’t associate with the entire PV? Or the PV is smaller? If we compare to previous figures looking at how LAMP1 outlined the PV, the PV looks to be a different shape here? An explanation/interpretation of this staining difference would be helpful

On the figure, for Latex beads, no drug, DAPI staining, a star is used, but there isn’t an explanation for what it denotes in the figure legend, nor is there an explanation for the arrows. Two of the labels for “PI(3,4)P2” are missing the comma.

See notes for “Figure Formatting.” Can each individual point be added to 3B as seen in Fig 1C?*

Response: We thank the Reviewer for comments on the role of phosphoinositides in this infection, which we discussed in Figure 3. As stated earlier, we have changed the discussion on phosphoinositides in this manuscript. The distribution of phosphoinositides in *Leishmania* infections is a topic that my laboratory has previously published on. In this manuscript, we present new data using biosensors to confirm that PI4P and PI(3,4)P2 are displayed on LPVs. That said, the issue of the morphological distribution of phosphoinositides on LPVs is worthy of discussion. In experiments that necessitate permeabilization, some solubilization of lipids may occur, which could alter their distribution. This is a topic that we will return to in subsequent studies.

*Reviewer 3: Figure 4
The labeling in the text says the western blots for the KD of TSG101 and ALIX are 4B, and selected lines are shown in 2C? But that is not consistent with the actual figure numbering. Overall, these experiments are clear and the data supports the conclusion.*

*See notes for “Figure Formatting.” Can each individual point be added to 4B-G as seen in Fig 1C?*

Response: The references to the Figures in the text have been corrected. The Figures have been re-drawn as suggested by the Reviewer.

*Reviewer 3. Figure 5*

*The images used here, with the arrows and cut outs showing the zoom ins, and the text description, are extremely effective, and this same approach should be taken with earlier images as well to point out the key features, especially in figure 1. But with the colors changed to white for the individual channels and colors only in merge (as stated in the “Figure Formatting” note). This explanation of the results and conclusions in this section are very clear. The diagrams are stretched, and the text is too small to read, but they are effective, so corrected formatting would fix. If diagrams like this could be added to figure 1 and 2, that would really improve the readers ability to follow along.

Can the statistical analysis on part C be added to the figure legend? Also each individual point, as seen in Fig 1C.*

Response: We thank the Reviewer for the complimentary statements about this figure (Figure 7 in the revised manuscript). Infections in the THP-1 are much clearly delineated and can therefore be presented with greater clarity. As mentioned earlier, the data from RAW264.7 cells has been streamlined to avoid confusion for the reader.

*Reviewer 3. Figure 6
It is stated that the number of LdLPVs is fewer in ALIXKD cells, but there is no quantification/measurement presented. I recognize that may be difficult, given the disruption of LAMP1 signal. It seems that the disruption of LAMP1 distribution is more extreme in the THP-1 cells than the RAW macrophages used in Figure 4. Can you comment on that? Otherwise, this data is clear, the conclusions are well supported.

See notes in the “Figure Formatting” Section. Can each individual point be added to each graph as seen in Fig 1C?*

Response: We thank the Reviewer for raising this issue. The interactions of parasites with mouse and human cell lines are intriguing. In the experiments discussed here, both the RAW264.7 mouse macrophage cell line and the human THP-1 cell line were evaluated in large part to capture differences between these lines. The human cell line does not replicate after it is differentiated into the macrophage form. We find this characteristic to be quite useful.

*Reviewer 3. Figure 7

Need to add the mention of Fig 7C into the text of the paper (the experiment is mentioned, but not the words “Figure 7C”). This experiment is key to showing the survival of the parasite but lack of PV division, nice experiment.

See notes in the “Figure Formatting” Section.*

Response*:* We thank the Reviewer for this suggestion. The text has been modified accordingly.

Discussion

*Reviewer 3. “Together, these results show that ALIX plays a central role in recruiting the ESCRT machinery to LdLPVs where it catalyzes the scission of this of the LdLPV pseudo-organelle. That a non-canonical activation of the ESCRT machinery plays a role in the division of LdLPVs is a new function for the ESCRT machinery.”
I think this conclusion is well supported by the data presented, but there are a few grammatical errors. The additional “of this” in the first sentence. The second sentence is incomplete. Potentially rephrase to “This non-canonical activation of the ESCRT machinery playing a role in the division of LdLPVs is a new function for the ESCRT machinery.”*

Response: We thank the Reviewer for bringing these grammatical errors to our attention. The phrase in question has been changed.

*Reviewer 3. To repeat my earlier point from figure 3, which perhaps could be included in the discussion rather than the results, how is LLOMe-mediated damage repaired? Does the ESCRT machinery associate and pinch off the damaged membrane to be sent to the plasma membrane? In which case, you’d expect to see CHMP4B on the outside of the PV, not inside. Do you have any explanation for what CHMP4B could be doing, and how it is getting inside the PV?*

Response: We thank the Reviewer for their comments on the value of the experiments with LLOMe. As discussed above, we have included a statement in the discussion that presents references discussing the LLOMe in greater depth and insight.

Reviewer 3 “*Based on our observations, we suggest that ESCRT components are recruited constitutively to LdLPVs and are anchored to phosphoinositides displayed on the LdLPV membrane.”
I agree with this point, and the following explanation in this paragraph. It is a more nuanced explanation of this data than the initial conclusion stated at the start of the discussion. However, I don’t understand this statement:
“The parasite plays a role in synthesizing the phosphoinositide displayed on the LdLPV membrane.”
Which role do you mean? I interpret this to mean that the parasite itself is synthesizing phosphoinositides that are transferred to the PV membrane? Or that parasite kinases are phosphorylating the PIs on the PV membrane? Which hasn’t been addressed in any data in this paper. Please clarify.*

Response: We thank the Reviewer for the careful and thorough evaluation of the manuscript. The Kima laboratory has published previously on phosphoinositides in Leishmania infections. We have shown that both the PI(3,4)P2 and one precursor (PI4)P are displayed on LPV. It is not known what the source of the enzyme that catalyzes this conversion. In the paragraph highlighted by the Reviewer, we raise the possibility that a parasite-derived enzyme could also be in the mix. As I have stated previously, we are very interested in the biogenesis and distribution of phosphoinositides in Leishmania infections.

*Reviewer 3. You state that the graphical abstract is “highlighting that when ALIX levels are limiting, LdLPVs are large and can harbor 3- 10 parasites”, I don’t think that’s clear in the diagram. Are you using the text size to indicate the amount of ALIX? Maybe you could use [ALIX] or a small up or down arrow?*

Response: We thank the Reviewer for the suggestion. We have updated to graphical abstract and included up and down arrows.

*Reviewer 3. It’s interesting that the prevention of the PV separation prevents the eventual dispersal of the parasite to new cells. Given that L. amazonensis has a communal PV for multiple parasites, can you elaborate on how you suspect these pathways would be used in L. amazonensis? Would they still require ESCRT machinery? If the parasites still drive expression of ALIX, it could suggest other roles for ALIX, or ESCRT machinery generally, outside of PV division*.

Response: This is a fascinating aspect of these studies. We thank the Reviewer for raising this issue. We should note that Reviewer 1 raised this issue as well. In new experiments that we shared in the Response to Reviewer 1, we found that *L. amazonensis* replication in THP-1 cells was not altered in ALIXKD as compared to TSG101KD of the shCTRL. Vacuole sizes were also unaffected. As we mentioned, we have elected not to make firm conclusions because we haven’t conducted experiments in RAW264.7 cells. It will be of interest to determine in greater detail how Leishmania species differentially engage the ESCRT machinery.
