# Supplementary material for: A non-canonical activation of the host’s ESCRT machinery is required for the scission of parasitophorous vacuoles and the replication of Leishmania donovani
Source: PLoS Pathog. 2025 Sep 16;21(9):e1013513. doi: 10.1371/journal.ppat.1013513 (PMC12453208; doi:10.1371/journal.ppat.1013513)
Supplement: Attachment — Submitted filename: Respnose to Reviewers _ 2.docx [file ppat.1013513.s012.docx]

Institute of Food and Agricultural Sciences

**Department of Microbiology and Cell Science**

**Building 981 Museum Road, Post Office Box 110700**

**Gainesville, FL 32611-0700; Tel: 352-262-1569; Fax 352-392-5922**

We thank the Editor and the Reviewers of our manuscript for their kind words about the suitability of our manuscript for publication.

What follows now are specific responses to the Reviewers' comments.

*Reviewer #2 wrote: The authors have a very nice manuscript, and the work moves the field forward. The authors need to be much more specific as to how they score a LdLPV. How is this assessed (see below)? Some of the quantification seems to be from subjective calls of association. There is not a clear description of how you are doing your image analysis. If it is scored by hand and not an automated analysis the parameters being used as criteria for the assessment needs to be very well described.*

Response: We thank the Reviewer for acknowledging that our work moves the field forward. Concerning the issue of how LdLPVs are scored and the likelihood that ‘quantification seems to be from subjective calls of association’, we would like to draw the Reviewers' attention to the section titled ‘Image Analysis’ in the Materials and Methods section. Specifically, we wrote: ‘Images were analyzed using the BZ-X800 analyzer for pictures taken in the BZ-X810 fluorescent microscope, or Zen Blue 2.6 for pictures taken in the Zeiss Axio observer Z1/7. Z stack projections were used for specific image analysis of colocalizing proteins. Using Fiji/ImageJ, a mask was created around parasite vacuoles or latex bead phagosomes determined to be regions of interest (ROI). Then, the BIOP JAcOP colocalization of signals plug-in was used to determine if the signals of proteins of interest colocalized in the ROI previously selected using the masking option. Lamp1 reactivity was used to delineate the vacuole of the parasite or the latex bead phagosome; colocalization of Lamp1 with ESCRT proteins of interest was performed. If the Pearson's correlation coefficient yielded by JACoP met a specific threshold for the masked region, then it was counted as positive. At least 100 parasite vacuoles or latex bead phagosomes were counted per coverslip, per treatment and timepoint. This analysis was applied for each experimental scheme.)’

We will say more below.

Reviewer #3 wrote: *This study evaluates the recruitment of the host cell ESCRT machinery to the Leishmania donovani parasitophorous vacuole. When Ld divide, the parasitophorous vacuole must divide as well, and this study shows compelling evidence of he role of ESCRT machinery, specifically ALIX, playing a role in this division.This study explores an interesting question of ESCRT recruitment, and they mention the implications of this work in other intracellular pathogens. The response of the authors to the reviewers was convincing, and the revised manuscript has simplified the experiments to provide a more streamlined story. The figures are more clear and effective than the first draft. There still remains a few typos that result from the shuffling of figures (ie. the data of 1D is referred to as 1C in a few spots), but overall this paper should be accepted.*

Response: We thank the Reviewer for this accurate summary of the biology of Leishmania parasites in macrophages and for affirming that our results are convincing. On the issue of typos, it appears that the version of the edited manuscript that was sent to the Reviewers wasn’t the ‘finalized’ edited manuscript. Nonetheless, in this submission, we have made every effort to ensure that there are no ‘typos’.

.

*More comments from Reviewer #2: Fig 1B Pearson's correlation coefficients should be reported for colocalization with LAMP1*

*Fig 1C The Axis should be labeled as LAMP1 since not every structure is parasitophorous vacuole. In all figures the axis label should be more definitive. It is not clear how you define structures.
Fig 1D In this example cell there is only a single phagosome. In the Leishmania vacuoles (not shown here) there are more units per cell than the latex beads, is this considered in the quantification? Are you using all of the LAMP1 signal as your denominator? Basically, there is not enough clarity on the scoring metrics used. It would only be fair to quantify the LAMP1 signal within a certain distance of the bead or Leishmania, and then to score the CHMP2B/4B on that compartment.
Fig1F Again Pearson’s CC should be done and on both infected and non-infected*.

Response: We thank the Reviewer for bringing their concerns about these Figures to our attention. Please note that the labeling of the figures has been updated in the finalized document that was uploaded to the Journal website. That said, we will proceed to address the concerns of the Reviewer.

Interest in scoring molecules recruited to structures within cells has led to the development of image analysis plugins, including the BIOP JAcOP plug-in, which we utilized for our analysis (https://imagej.net/plugins/jacop). Briefly, we created a cell mask around the phagosome that contains the parasite or the latex bead, thereby excluding signals from late endosomes and lysosomes from the statistical analysis. By creating this cell mask around the phagosome, one can use the BIOP JAcOP plug-in of FIJI to measure how many pixels of both signals colocalize, specifically in the region of interest (ROI), excluding other cytosolic or non-phagosome compartments. The BIOP JAcOP plug-in then calculates and reports a Pearson’s correlation coefficient for the signal of interest. In our analysis, a score of 0.4 (or 40% colocalization) was considered positive for colocalization. **Please note that the positive colocalization to LdLPVs that was plotted in the figures was determined by the Pearson correlation coefficient score**. At least 100 total phagosomes harboring parasites or latex beads (regardless of how many phagosomes are in each cell) were evaluated in each experiment.

*More comments from Reviewer 2: Collectively there is an issue with parsing out what is a vacuole verses a late endosome / lysosome. Please note that late endosomes and lysosomes have LAMP1 signal. Using the Leishmania DAPI signal the compartments can be scored. It would be ideal if in a supplemental figure a detailed analysis pipeline can be outlined with sample images highlighted to show the reader what is being scored. Is LAMP1 being scored as an LdLPV or do you require the DAPI signal within and LAMP1 compartment?
There is not very much said about the reticular morphology of the LAMP1 signal. Please add some notes on this in the early section of the results and comment on this morphology shift in the discussion. Is the LdLPV essentially the same as the Salmonella containing compartment you mention in the introduction? Please add a comment on this in the discussion*

Response:

On the issue of phasosome morphology, phagosomal compartments that carry parasites or latex beads can potentially have an infinite number of distinct shapes, making it almost impossible to generate a code that automates this process without potentially introducing bias (for example, the code might be accurate with distinct shapes while leaving more ambiguous shapes out of the statistical analysis since they may not be recognized as such). When you make a cell mask around the phagosome (Dapi label of parasite and label of latex bead), signals from late endosomes and lysosomes are left **OUT** of the statistical analysis.

We want to bring to the attention of the Reviewer other manuscripts that have employed the methods used here for scoring colocalization.

<https://www.embopress.org/doi/full/10.15252/embj.2020104494>;

(<https://www.embopress.org/doi/full/10.15252/embj.201899753>)

Considering that there are videos on YouTube that explicitly describe the assessment of colocalization of signals using the BIOP JAcOP plug-in, we don’t think that a supplemental figure is warranted here.

We have updated the description of the method used for image analysis in the Materials and Methods section. Here is the updated section

**Image Analysis**

Images were analyzed using the BZ-X800 analyzer for pictures taken in the BZ-X810 fluorescent microscope, or Zen Blue 2.6 for images taken in the Zeiss Axio observer Z1/7. Z stack projections were used for specific image analysis of colocalizing proteins. Using Fiji/ImageJ, a mask was created around parasite vacuoles or latex bead phagosomes, determined to be regions of interest (ROI). The DAPI signal of the parasites, as well as the blue fluorescence of the latex beads was used to determine the ROI. The purpose of creating an ROI mask around the phagosome of a parasite or bead is to exclude cytosolic or other compartmental protein signals from the colocalization analysis. Then, the BIOP JAcOP colocalization of signals plug-in was used to determine if the signals of proteins of interest colocalized in the ROI previously selected using the masking option. LAMP1 reactivity was used to delineate the vacuole of the parasite or the latex bead phagosome; colocalization of LAMP1 with ESCRT proteins of interest was performed. If Pearson's correlation coefficient, as yielded by JACoP, met a specific threshold of 0.4 or higher for the masked region, it was considered positive. At least 100 parasite vacuoles or latex bead phagosomes were counted per coverslip, per treatment, and time point. This analysis was applied for each experimental scheme.
